# Prevalence of Type 2 Diabetes and Prediabetes in the Gwalior-Chambal Region of Central India

**DOI:** 10.3390/ijerph16234708

**Published:** 2019-11-26

**Authors:** Senthil Kumar Subramani, Dhananjay Yadav, Meerambika Mishra, Umamaheswari Pakkirisamy, Prakesh Mathiyalagen, GBKS Prasad

**Affiliations:** 1 School of Studies in Biochemistry, Jiwaji University, Gwalior 474011, India; sskbio.03@gmail.com; 2 Department of Biotechnology, Tropilite Foods Pvt. Ltd., Davar’s Campus, Tansen Road, Gwalior-474002, India; 3 Department of Medical Biotechnology, Yeungnam University, Gyeongsan 38541, Korea; dhanyadav16481@gmail.com; 4 Department of Infectious Diseases and Immunology, College of Veterinary medicine, University of Florida, Gainesville, FL 32601, USA; meerambika.mishra@gmail.com; 5 Department of Pediatric Nursing, Shivnath Singh College of Nursing, Chirwai Naka, Shivpuri link road, Gwalior 474001, India; umaamu05@gmail.com; 6 Department of Community Medicine, Indira Gandhi Medical College and Research Institute, Puducherry 605 009, India; drmanivelprakash@yahoo.in

**Keywords:** epidemiology, type 2 diabetes, prediabetes, Gwalior-Chambal region, Central India

## Abstract

Aim: This study evaluated the prevalence of prediabetes and type 2 diabetes mellitus in the Gwalior-Chambal region of India. Methods: A cross-sectional house-to-house survey was conducted on a population of 7608 subjects, aged between 20 and 79 years for fasting blood glucose level in finger-prick blood. Participants were stratified based on blood glucose levels, gender, age, family history, etc. to assess their impact. Result: The prevalence of type 2 diabetes and prediabetes in the Gwalior-Chambal region was found to be 11.4% and 5.7%, respectively. The prevalence of diabetes was significantly higher in the urban population (12.7%) while that of prediabetes was higher in the rural population (7.9%). Male subjects recorded a higher prevalence of prediabetes (8.2%, OR 1.54 in rural; 5.1%, OR 1.26 in urban) as well as diabetes (rural 9.2%, OR 3.15; urban 16.5%, OR 1.57). Both prediabetes and diabetes were recorded as being higher in those subjects leading a sedentary lifestyle and in the aged population. The prevalence of hyperglycemia was much higher in those with a family history of type 2 diabetes (30.6% in rural, 21.5% in urban). Almost half of the diabetics in the rural population were diagnosed for the first time. The multivariate regression analysis identified male gender, increasing age of 30 years and above, and positive family history as significant risk factors for diabetes whereas age of 40 to 79 and less physical activity were significant risk factors for prediabetes. Conclusion: Family history of diabetes, and sedentary lifestyle appeared as key factors promoting prediabetes and diabetes in the Gwalior-Chambal region. A lack of awareness appeared as one of the major causes of the high prevalence in the rural region.

## 1. Introduction

Diabetes mellitus (DM) is a chronic metabolic disorder characterized by hyperglycemia resulting from defects in insulin secretion, insulin action, or in both [1]. Long term hyperglycemia leads to micro and macrovascular complications [2]. Diabetes mellitus occurs throughout the world but is more common (especially type 2) in the developing and developed countries [3].

The International Diabetes Federation has published a report, which stated that in 2017, 425 million people worldwide suffered from DM, and if this trend continues till 2045, a load of 629 million patients is extrapolated [4]. South Asian countries have undergone rapid developmental urbanization, resulting in changeovers in the nutritional statuses in recent decades. It has in part led to a high prevalence of DM. As compared with other ethnic groups, south Asian people tend to develop type 2 diabetes at a younger age. Furthermore, the south Asian phenotype is characterized by a predisposition to central deposition of fat on a small frame; this has been referred to as thin-fat or metabolically obese-normal weight. This phenotype is exacerbated in an increasingly obesogenic environment [5]. More than 60% of the world’s diabetic population resides in Asian countries [6]. India has a growth rate of 12.5%, and 20% of the world’s population is facing an increased risk of diabetes mellitus in its urban sectors [7,8]. Indeed, the prevalence of diabetes in India is expected to rise from 8.8% (in 2017) to 11.4% by 2045. However, around 60% of the people with diabetes go undiagnosed in the South-East Asian population [4]. South Asians with type 2 diabetes often have delayed diagnosis and inadequate management of glycaemia and other risk factors, leading to severe microvascular and macrovascular complications in this population [9,10]. Earlier, we reported the high prevalence of diabetes in metabolic syndrome subjects in the Gwalior-Chambal region of Central India [11,12,13]; however, no detailed study with a focus on the prevalence of diabetes mellitus has been done in this area. Hence, the present work aimed to study the prevalence of prediabetes and diabetes in the Gwalior-Chambal region of the northern part of Central India.

## 2. Methods

### 2.1. Study Design, Population, and Sample

A cross-sectional study was carried out in the urban (26°12′19.8″ N 78°11′57.9″ E) and rural (26°29′41.1″ N 78°36′52.6″ E, 26°0 26°00′29.6″ N 77°37′25.7″ E) areas of the Gwalior-Chambal region of Central India during May 2015 to September 2017. Subjects were selected as between 20 and 79 years of age and of either gender. The institutional human ethical committee approved this study (No. JU/IHEC/2013-A/08).

**Figure 1:**
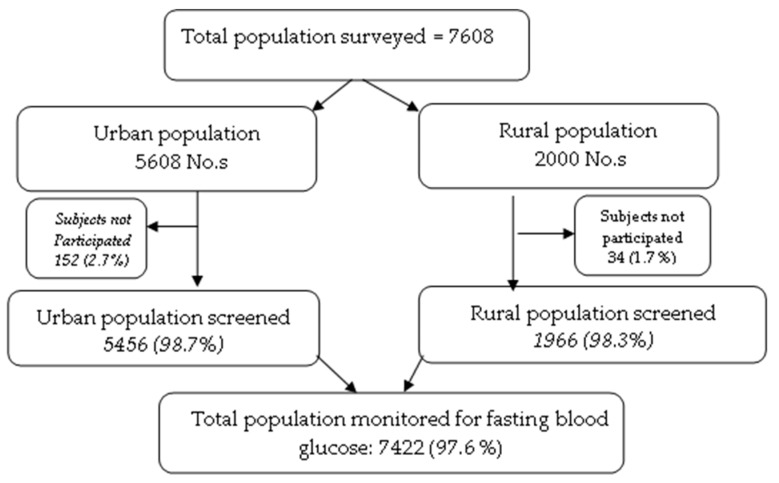
Outline of the population surveyed.

The non-probability convenience sampling method was chosen with the door-to-door approach to collect the samples. The subjects/households screened were given prior counseling regarding the purpose and benefits of the study. Also, subjects were advised to fast for a minimum of 10 h before blood sampling. As per the consent of the volunteers, their fasting blood glucose levels in finger-prick whole blood were measured using a Glucometer (Accu-Chek^®^ Roche Diagnostics GmbH, D-68298 Mannheim, Germany) (Figure 1). These subjects were in the 20–79 years age group with a mean age of 42.6 years (43 years in urban areas and 41.4 years in rural areas).

### 2.2. Data Collection

Anjana et al. [14] reported earlier that the Indian prevalence of type 2 diabetes was 9.4%; the sample size was calculated by using the formula 4pq/l^2^, where *p* = 9.4, q = 100 – p, and l is the relative error of 5% and 5% non-responders. So, the final target sample size was 7608 (Figure 1). The subjects were categorized into diabetes and prediabetes following WHO criteria [15]. Those with fasting blood glucose levels ≥126 mg/dL (i.e., ≥7.0 mmol/L) after an overnight fast were categorized as diabetic, and those between 110 and 125 mg/dL (i.e., 6.1–6.9 mmol/L) were considered as pre-diabetic. Further, demographical details and personal information about each subject, such as age, food habits, physical activity, and personal habits like smoking, alcohol consumption, and family history, were recorded one day before screening by using structured questionnaires (Table 1).

### 2.3. Data Analysis and Risk Prediction

The continuous variables are presented as means and standard deviations. The prevalence of diabetes and prediabetes was calculated in percentages, and the Clopper-Pearson confidence interval (95%) was used for the observed proportion and derived using MedCalc software. A receiver operating characteristic curve (ROC) analysis with the Youden index J statistic was analyzed to measure the optimal cut-off point of age for predicting the risk of developing prediabetes and diabetes. Statistical Package for the Social Sciences (SPSS) version 20 was used to calculate the risk ratio of every attributing factor to diabetes and prediabetes conditions. A *p*-value of less than 0.05 was considered statistically significant.

## 3. Results

### 3.1. Basic Characteristics of the Subjects

**Figure 2:**
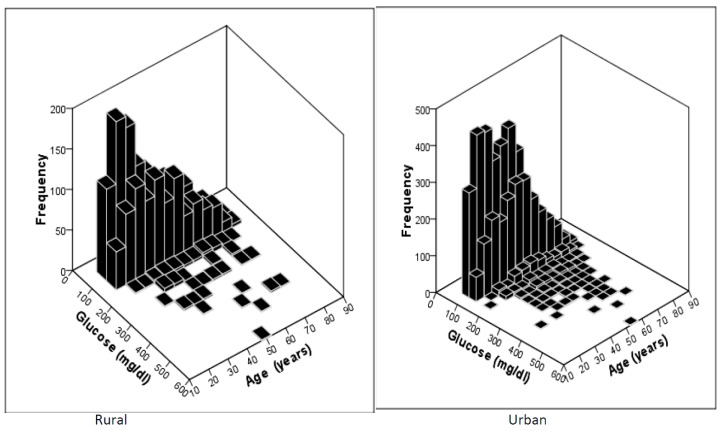
Frequency distribution of the study population based on age and blood glucose levels.

There were 7422 subjects in the selected households, covering both males (N = 3467) and females (N = 3955). Of these, 5456 subjects were from urban areas (males = 2429, females = 3027) and 1966 from rural areas (males = 1038, females = 928). Blood glucose was measured in these subjects during the fasting condition and categorized into normal, diabetes, and prediabetes based on the WHO criteria. The distribution of age and blood glucose values are shown in Figure 2. The mean age group of the study subjects was 42.1 ± 14.2 years; this was 42.64 ± 13 years, and 40.73 ± 14.7 years in urban and rural areas, respectively.

### 3.2. Prevalence of Diabetes and Prediabetes Based on Gender and Age

The mean prevalence of diabetes and prediabetes was found to be 11.4% and 5.7%, respectively. The prevalence was found to be 7.7% and 7.9% in rural areas, whereas in urban areas, it was 12.7% and 4.9%, respectively. Stratification of diabetics based on the gender among the male rural population and urban male population was found to be 9.2% and 16.5%, respectively. Prediabetes, on the other hand, was found to be in 8.2% of rural males compared to 5.1% of the urban population. A higher prevalence of diabetes was observed in the age group of 60–69 years. However, in the rural population, both diabetes and prediabetes were recorded as high in the 70–79 age group (Table 2).

### 3.3. Prevalence of Diabetes and Prediabetes Based on Physical Activity and Occupation

Further categorization of the study population was done based on their daily physical activity. The prevalence of diabetes was 8.9% among less physically active people in the rural region. The prevalence was recorded as higher, i.e., 13.4%, even among moderately active subjects in the urban populations. Based on occupation, rural laborers recorded a diabetes prevalence of 9.7% while in the urban population, the employees showed a higher prevalence of diabetes (16.3%). In rural areas, the prevalence of prediabetes was higher in employees (9.1%), and in urban areas, this prevalence was 6.1% in household workers (Table 2).

### 3.4. Prevalence of Diabetes and Prediabetes Based on Family History and Personal Habits

The prevalence of diabetes was significantly higher in those with a family history of diabetes, i.e., 30.6% in the rural population and 21.5% in the urban population (Table 2).

The prevalence of diabetes and prediabetes was also stratified based on personal habits, such as smoking and alcohol drinking. In the rural population, smoking and drinking alcohol showed a positive association with a high prevalence of diabetes, i.e., 9.6% and 10.8%, respectively. The prevalence of diabetes (PD) and prediabetes (PPD) were analyzed based on the nature of food consumed. People on a vegan diet showed a significantly higher prevalence (21.1% in the rural population) (Table 2). About half of the diabetic populations (49.3%) were diagnosed for the first time in rural areas (Table 2).

**Figure 3:**
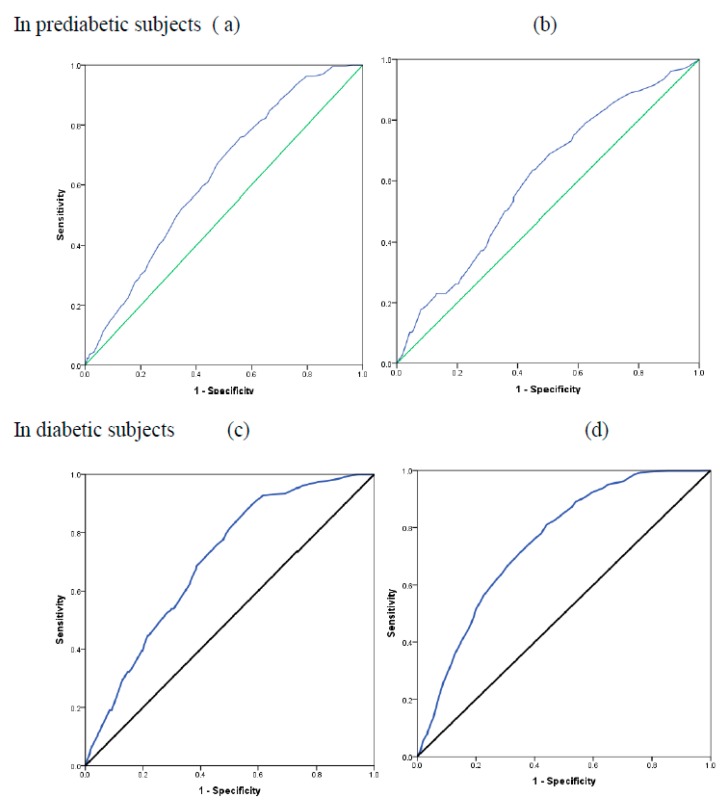
Receiver operating characteristic analysis to determine the optimal cut-off values of the age to predict the risk of developing pre diabetes and diabetes in urban (**a**,**c**) and rural (**b**,**d**) areas.

The receiver operating characteristic analysis is shown in Figure 3 to estimate the optimal cut-off values of age to predict the risk of developing prediabetes and diabetes in urban (a,c) and rural (b,d) areas. Age has a statistically significant role in predicting prediabetes in urban and rural areas, with a cut-off of 40.5 and 36.5 years, respectively. In diabetes, in urban and rural areas, a cut-off of 45.5 and 39.5 years, respectively, was observed. In prediabetes subjects, the urban area noted 67.17% and 52.27% sensitivity and specificity, while in rural areas, it was 66.67% and 51.21%. In diabetic subjects, the urban areas noted 71.22% and 65.04% sensitivity and specificity; on the other hand, in rural areas, it was 75% and 55% (Figure 3, Table 3).

Multinomial regression analysis of both the areas between various groups showed the following tendencies. In rural areas, normal males (OR 3.15 (95% CI: 1.18–8.44)) in the age group 70–79 years (OR 8.01 (95% CI: 3.26, 19.70)), with a family history of diabetes (OR 7.66 (95% CI: 3.91–14.97)) were significantly associated with diabetes. However, in urban areas, normal males (OR 1.57 (95% CI: 1.22–2.03)) in the age group 60–69 years (OR 20.25 (95% CI 11.88–34.52)), with a family history of diabetes; (OR 2.99 (95% CI 2.45–3.64)) were significantly associated with diabetes. In prediabetes, in rural areas, those aged 70 to 79 years (OR 5.32 (95% CI: 2.49–11.33)) were at a significantly higher risk. Whereas, in the urban population, this was true for those aged 60 to 69 years (OR 4.20 (95% CI: 2.24–7.87)), with a family history of diabetes (OR 1.41 (95% CI: 1.03–1.95)). Physical activity and personal habits did not show any statistically significant associations in both the rural and urban study population (Table 4).

## 4. Discussion

A cross-sectional study was conducted to determine the prevalence of diabetes as a rising problem in the Gwalior-Chambal region of northern Central India. The present study assessed the prevalence of fasting hyperglycemia in urban as well as rural zones covering all age groups and major professions. House-to-house visits in randomly selected localities educated subjects a day before blood sampling and obtained confirmation at the time of blood sampling on the subjects’ fasting state, ensuring the blood samples monitored were truly fasting in nature. Type 2 diabetes was found to be significantly higher (11.4%) than prediabetes (5.7%). Males recorded a relatively higher rate of diabetes (14.3%) than female counterparts (8.9%) and no such gender variation was recorded in the case of prediabetes irrespective of their domicile. The recent report by Anjana et al. also showed a high prevalence in the male population [16]. The higher prevalence of type 2 diabetes in men than women was associated with a larger amount of visceral fat in men. In contrast, differences in body mass index were not associated with this difference [17].

The present study recorded a prevalence of 7.7% for diabetes in rural areas, which is higher than earlier reported estimates (6.4%) [14]. Two studies conducted earlier in other rural parts of Madhya Pradesh State reported 33.7% and 14.5% diabetes prevalence [18,19]. However, both of the above studies were clinical-based reports. Urban diabetes recorded in our study in the Gwalior-Chambal region was 12.7%, a relatively lower figure than the 15.2% reported by Khan et al. in the neighboring urban Bareilly region [20]. However, it was higher than the recent 11.2% estimate in 15 states of India [16].

Our previous reports have suggested that the prevalence of dyslipidemia, hypertension, and clusters of metabolic factors are higher in type 2 diabetes, and therefore we can say that increasing the proportion of these metabolic factors can lead to an increased prevalence of diabetes in Central India [11,12,13]. The other plausible reason for the high prevalence of diabetes in an urban area might be air pollution. According to earlier reports, Gwalior is one of the most air-polluted cities in India [21,22,23]. The recent study reported that the prevalence of diabetes was higher among people living in areas more highly exposed to PM_2.5_ (air particulate matter 2.5) compared to those living in areas with lower exposures to PM_2.5_ [24]_._ The exact reason for the association between air pollution and a higher prevalence of diabetes is not completely understood. Moreover, we did not have exact data that could relate particulate exposure with diabetes prevalence in our studied region.

Based on the ROC analysis, the rural population is at risk of prediabetes and diabetes at an earlier age compared to the urban population (Table 3). The older population aged above 60 years recorded the highest rate of diabetes (27.6% in the urban area). Our data corroborate with the earlier observations of Corsi and Subramaniyum [25]. Older patients are more likely to present cardiovascular complications and comorbid conditions [26]. The high incidence in older age groups could be attributed to poor immunity and lesser physical activity, although they are not stressed professionally. The pervasiveness of diabetes was significantly associated with a prior family history as expected. The higher prevalence of diabetes in adult urban groups than their rural counter parts is attributed to a stressful lifestyle. The high incidence of diabetes in those with a family history of diabetes further confirms the genetic basis of this metabolic disorder. Arora et al. [27] and Ahmad et al. [28], in their studies, also noted a significant association between a family history and high incidence of diabetes.

The study recorded two interesting observations, including a high prevalence of hyperglycemia among rural agricultural workers and vegetarians. The rural folk lifestyle is active physically but may be poor nutritionally. Thus, this study sheds light on the importance of nutrition and its possible association with the quality of nutrition. Drewnowski and Specter reported that people who eat less healthy diets suffer from the highest rates of obesity and type 2 diabetes [29].

Other reports indicate that treatment of T2DM prevents patients from developing early end-organ complications, which can be achieved through proper dietary management. Patients should also have good knowledge about the disease and diet. Active and effective dietary education may prevent the onset of diabetes and its complications [30].

Studies state that acute exercise improves the immune system and metabolic health, including an observed inverse relationship between moderate exercise training and illness risk [31]. In this study, we observed a diabetes prevalence of 8.9% among less physically active people in the rural region. The prevalence was recorded as higher i.e., 13.4%, even among moderately active urban populations. Colberg et al. reported that physical activity and exercise should be recommended to all individuals with diabetes in the course of management of glycemic regulation [32].

Personal habits appear to be insignificant in regard to diabetes. However, there are studies linking diabetes with smoking [33] and alcohol consumption [34]. Earlier cohort studies have shown that light and moderate alcohol consumption was associated with a lower risk of T2D, whereas heavy alcohol consumption was not related with the risk of T2D [35]. The high incidence of diabetes in vegetarians finds no convincing answers unless the individual of this group has a family history coupled with a sedentary lifestyle and/or are subjected to a stressful life. Eating only vegetables does not necessarily relate to good nutrition, because if these “vegetables” are composed primarily of foods with a high glycemic index that are low in fiber and other nutrients and non-nutrients, increased intake could be harmful to health and increase the risk of diabetes [36]. Another possibility is that the vegetables consumed may contain high amounts of pesticide/herbicide residues, which might trigger diabetic circuits in the body. A recent report shows that the occurrence of diabetes among farmers was associated with pesticide exposure [37]. Fast food with processed carbohydrates, such as bread, noodles, and cornstarch, high-calorie drinks, and vegetable fat contributes greatly to urban diabetes [38]. Meyer et al. found that vegetable fat (saturated fats) intake remained a significant predictor of new diabetes [39]. More than half of the world’s population remains undiagnosed for diabetes [40]. About half (49.3%) of the diabetic population, particularly in rural areas, weree diagnosed for the first time and this fact denotes the necessity of periodic diabetic surveys among the population. Our study has few limitations, including that the diabetes prevalence was assessed based purely on fasting hyperglycemia. A considerable number of prediabetics exhibit postprandial hyperglycemia, which is not feasible in field-oriented studies of this kind. Also, glycosylated hemoglobin levels, the main criteria for diabetes detection, could not assessed due to practical problems in the filed study. Furthermore, a causal relationship could not be established because of the cross-sectional study design.

## 5. Conclusions

Diabetes prevalence was found to be significantly high in older age groups and the male population. The incidence of prediabetes is alarmingly increasing diabetes risk in this area. Those with a family history of diabetes coupled with a sedentary lifestyle are at a high risk of developing diabetes. Thus, the predisposing factors to diabetes include a sedentary lifestyle, professional stress, and prior family history. The study underlines the importance of diabetic education to vulnerable groups of the society, particularly to prediabetics and diabetics, to protect them from serious complications associated with diabetes mellitus.

## Figures and Tables

**Table 1:** Stratification of the study population based on various criteria.

Characteristics	Subgroup	Rural				Urban			
		Frequency	%	95% CI		Frequency	%	95% CI	
				Lower	Upper			Lower	Upper
Gender	Male	1038	52.8	50.56	55.02	2429	44.5	43.17	45.83
	Female	928	47.2	44.97	49.43	3027	55.5	54.17	56.82
	Total	1966	100			5456	100		
Age	20–29	541	27.5	25.53	29.53	1205	22.1	21.0	23.22
	30–39	467	23.8	21.93	25.74	1085	19.9	18.8	20.98
	40–49	359	18.3	16.61	20.08	1432	26.2	25.0	27.38
	50–59	305	15.5	13.92	17.17	991	18.2	17.1	19.25
	60–69	190	9.7	8.42	11.09	527	9.7	8.92	10.51
	70–79	104	5.3	4.35	6.3	216	4.0	3.50	4.55
	Total	1966	100			5456	100		
Physical activity	Less	1082	55.0	52.77	57.22	1225	22.5	21.39	23.63
	Moderate	629	32.0	29.94	34.11	3744	68.6	67.35	69.83
	More	255	13.0	11.54	14.57	487	8.9	8.15	9.68
	Total	1966	100			5456	100		
Family history of diabetes	Without FHD	1904	96.8	95.92	97.53	4443	81.4	80.34	82.42
	With FHD	62	3.2	2.46	4.07	1013	18.6	17.57	19.65
	Total	1966	100			5456	100		
Food habits	Vegetarian	1895	96.4	95.47	97.17	4280	78.4	77.28	79.48
	Both	71	3.6	2.82	4.52	1176	21.6	20.51	22.71
	Total	1966	100			5456	100		
Occupation	Labor	751	38.2	36.05	40.39	237	4.3	3.78	4.87
	Office Job	232	11.8	10.41	13.31	2088	38.3	37.1	39.61
	Household work	758	38.6	36.44	40.73	2162	39.6	38.29	40.91
	Others	225	11.4	9.93	12.78	969	17.8	16.79	18.84
	Total	1966				5456			
Personal habits	Non smokers	1634	83.1	81.36	84.73	5119	93.8	93.12	94.42
	Smokers	332	16.9	15.26	18.63	337	6.2	5.57	6.8
	Total	1966	100			5456	100		
	Non-alcohol consumer	1846	93.9	92.75	94.91	5173	94.8	94.17	95.37
	Alcoholics	120	6.1	5.08	7.25	283	5.2	4.62	5.82
	Total	1966	100			5456	100		

**Table 2:** Diabetes and prediabetes among the study population in the Gwalior-Chambal region.

Characteristics	Subgroup	Rural							Urban						
		Total	Diabetes			Prediabetes			Total	Diabetes			Prediabetes		
				95% CI			95% CI				95% CI			95% CI	
			% (N)	Upper	Lower	% (N)	Upper	Lower		% (N)	Upper	Lower	% (N)	Upper	Lower
	Total	1966	7.7 (152)	8.97	6.56	7.9 (156)	9.18	6.75	5456	12.7 (695)	13.61	11.83	4.9 (265)	5.51	4.34
Gender	Male	1038	9.2 (95)	11.1	7.5	8.2 (85)	10	6.6	2429	16.5 (401)	18	15	5.1 (123)	6	4.3
	Female	928	6.1 (57)	7.8	4.7	7.7 (71)	9.6	6	3027	9.7 (294)	10.8	8.7	4.7 (142)	5.5	4
Age	20–29	541	1.8 (10)	3.31	0.86	4.6 (25)	6.72	3	1205	0.8 (10)	1.35	0.31	1.7 (23)	2.60	1.05
	30–39	467	6 (28)	8.55	4.02	6.9 (32)	9.59	4.77	1085	7.3 (79)	8.02	5.02	4.1 (50)	5.46	3.00
	40–49	359	9.7 (35)	13.24	6.84	11.4 (41)	15.15	8.3	1432	12.4 (178)	15.08	12.15	6.6 (85)	8.01	5.37
	50–59	305	11.1 (34)	15.17	7.8	7.5 (23)	11.05	4.81	991	21.8 (216)	26.99	21.56	6.9 (62)	8.66	5.40
	60–69	190	15.3 (29)	21.22	10.5	10 (19)	15.18	6.13	572	27.6 (158)	36.2	28.38	6.7 (33)	9.07	4.79
	70–79	104	15.4 (16)	23.8	9.07	15.4 (16)	23.8	9.07	261	20.7 (54)	32.29	21.25	5.9 (12)	9.49	3.37
Occupation	Labor	751	9.7 (73)	12.4	7.9	8.1 (61)	10.12	6	237	6.8 (16)	12.8	4.16	5.4 (10)	9.98	2.51
	Job	232	7.3 (17)	11.3	4.1	9.1 (21)	13.98	5.93	2088	16.3 (340)	17.7	14.58	4.9 (107)	5.89	4
	HHW	758	7.3 (55)	9.39	5.55	8 (61)	10.1	6.2	2162	11.3 (244)	12.7	10	6 (129)	7.09	5.04
	Others	225	3.1 (7)	5.79	1	5.8 (13)	9.86	3.19	969	9.8 (95)	11.23	7.43	2 (19)	3.1	1.2
Physical activity	Less	1082	8.9 (96)	10.76	7.27	6.7 (72)	8.36	5.28	1225	11.4 (140)	13.31	9.67	3.4 (42)	4.57	2.46
	Moderate	629	5.7 (36)	7.81	4.02	8.9 (56)	11.4	6.79	3744	13.4 (503)	14.53	12.32	5.3 (197)	6.07	4.60
	More	255	7.8 (20)	12.11	4.65	11 (28)	15.8	7.23	487	10.7 (52)	13.79	8.10	5.3 (26)	7.68	3.49
FHD	No	1904	7 (133)	8.2	5.9	7.9 (150)	9.2	6.7	4443	10.1 (447)	11.7	9.8	4.8 (213)	5.5	4.2
	Yes	62	30.6 (19)	43.6	19.5	9.7 (6)	19.9	3.7	1013	21.5 (218)	24.2	19	5.1 (52)	6.6	3.8
Smokers	No	1634	7.3 (120)	8.67	6	8 (130)	9.4	6.7	5119	13 (663)	14	12.1	4.8 (245)	5.42	4.2
	Yes	332	9.6 (32)	14.53	7.59	7.8 (26)	11	5.15	337	9.5 (32)	13.2	6.59	5.9 (20)	8.98	3.6
Alcohol	No	1846	7.5 (139)	8.8	6.34	7.9 (145)	9.2	6.7	5173	12.9 (669)	13.8	12	4.8 (246)	5.42	4.2
	Yes	120	10.8 (13)	18.01	6.02	9.2 (11)	16	4.69	283	10.2 (29)	13.2	6.1	6.7 (19)	10.3	4.1
Food	Veg	1895	7.2 (137)	8.46	6.08	8.1 (153)	9.42	6.91	4280	13.3 (571)	14.4	12.3	4.9 (210)	5.59	4.27
	Both	71	21.1 (15)	32.4	12.31	4.2 (3)	11.8	0.87	1176	10.5 (124)	12.4	8.81	4.7 (55)	6.07	3.56
DS	Known		50.7 (77)							72.7 (505)					
	FTD		49.3 (75)							27.3 (190)					

Note: HHW = Household works, FDH = Family history of diabetes, Veg = vegetarian diet DS = diabetes status, FTD = First time diagnosed.

**Table 3:** Diagnostic value of age to predict the risk of developing prediabetes and diabetes in urban and rural areas.

Diagnostic Parameters	Prediabetes				Diabetes			
	Urban Area		Rural Area		Urban Area		Rural Area	
Area Under the Curve (95% CI)	0.630 (0.600–0.661)		0.607 (0.562–0.651)		0.747 (0.729–0.764)		0.701 (0.664–0.739)	
*p* value	<0.001		<0.001		<0.001		<0.001	
Cut off value	40.5 years		36.5 years		45.5 years		39.5 years	
Sensitivity	67.17%	(61.31, 72.54)	66.67%	(58.95, 73.59)	71.22%	(67.75, 74.46)	75%	(67.56, 81.21)
Specificity	52.27%	(50.81, 53.73)	51.21%	(48.8, 53.61)	65.04%	(63.63, 66.42)	55.07%	(52.66, 57.45)
Positive Predictive Value	7.659%	(6.646, 8.812)	11.39%	(9.49, 13.62)	23.95%	(22.16, 25.83)	13.27%	(11.17, 15.7)
Negative Predictive Value	96.43%	(95.62, 97.1)	94.23%	(92.51, 95.57)	93.6%	(92.68, 94.4)	96%	(94.56, 97.08)
Diagnostic Accuracy	53.1%	(51.68, 54.51)	52.54%	(50.23, 54.83)	65.86%	(64.56, 67.14)	56.74%	(54.45, 59.01)
Likelihood ratio of a Positive Test	1.407	(1.398–1.416)	1.366	(1.35–1.383)	2.037	(2.031–2.043)	1.669	(1.655–1.683)
Likelihood ratio of a Negative Test	0.6281	(0.6136–0.6429)	0.651	(0.6255–0.6775)	0.4425	(0.438–0.447)	0.454	(0.4304–0.4789)
Diagnostic Odds	2.364	(1.794–3.114)	2.099	(1.484–2.968)	4.604	(3.863–5.486)	3.677	(2.515–5.374)

**Table 4:** Multinomial regression analysis of study subjects.

Prediabetes					Diabetes			
Rural			Urban		Rural		Urban	
	AOR	95% C I	AOR	95% C I	AOR	95% C I	AOR	95% C I
Male	1.54	0.73–3.27	1.26	0.85–1.88	3.15	1.18–8.44 *	1.57	1.22–2.03 **
30–39 years	1.66	0.90–3.08	1.87	1.07–3.26 **	2.73	1.24–6.03 *	3.11	1.84–5.28 **
40–49 years	3.08	1.69–5.60 **	3.48	2.04–5.96 **	4.72	2.16–10.30 **	6.60	3.96–10.99 **
50–59 years	2.07	1.06–4.02 *	4.14	2.37–7.22 **	5.05	2.30–11.10 **	12.77	7.66–21.31 **
60–69 years	3.23	1.60–6.54 **	4.20	2.24–7.87 **	7.50	3.34–16.83 **	20.25	11.88–34.52 **
70–79 years	5.32	2.49–11.33 **	3.32	1.51–7.29 **	8.01	3.26–19.70 **	14.33	7.90–25.99 **
Office jobs	1.45	0.82–2.54	1.14	0.53–2.47	0.914	0.50–1.68	1.50	0.81–2.78
Household works	1.60	0.69–3.70	1.80	0.76–4.23	2.50	0.91–6.83	1.63	0.84–3.17
Others	1.4	0.61–3.40	0.56	0.19–1.84	0.46	0.96–2.19	0.07	0.009–0.60
With FHD	1.60	0.64–3.96	1.41	1.03–1.95 *	7.66	3.91–14.97 **	2.99	2.45–3.64 **
Smokers	0.82	0.51–1.33	0.88	0.44–1.77	1.06	0.67–1.68	0.63	0.38–1.04
Alcoholics	0.95	0.47–1.90	2.02	0.97–4.18	0.89	0.44–1.80	1.27	0.72–2.23
Vegetarian food	2.08	0.63–6.83	0.93	0.67–1.29	0.31	0.16–0.60 **	0.94	0.75–1.18
Less PA	0.57	0.35–0.92 *	0.60	0.35–1.02	1.82	1.02–3.24 *	1.08	0.74–1.57
Moderate PA	0.81	0.48–1.37	0.88	0.56–1.39	1.21	0.64–2.30	1.06	0.76–1.48

*p* value * = *p* < 05, ** = *p* < 001, AOR = adjusted odd ratio, FHD = Family history of diabetes, PA = physical activity.
